# Association of Antiretroviral Therapy with Platelet Function and Systemic Inflammatory Response in People Living with HIV: A Cross-Sectional Study

**DOI:** 10.3390/microorganisms11040958

**Published:** 2023-04-06

**Authors:** Karolina Akinosoglou, Martha Kolosaka, George Schinas, Anne-Lise Delastic, Stefania Antonopoulou, Angelos Perperis, Markos Marangos, Athanasia Mouzaki, Charalambos Gogos

**Affiliations:** 1 Department of Internal Medicine, University General Hospital of Patras, 26504 Patras, Greece; mkolosaka@gmail.com (M.K.);; 2 Medical School, University of Patras, 26504 Patras, Greece; 3 Division of Infectious Diseases, Department of Internal Medicine, University of Patras, 26504, Patras, Greece; 4 Laboratory of Immuno-Hematology, Medical School, University of Patras, 26504 Patras, Greece; 5 Department of Cardiology, University General Hospital of Patras, 26504 Patras, Greece

**Keywords:** HIV, platelets, inflammatory response, cytokines, P2Y12, GPIIb/IIIa, antiretroviral therapy

## Abstract

People living with HIV (PLWHIV) present an increased risk of adverse cardiovascular events. We aimed to assess whether antiretroviral therapy (ART) pharmacologically enhances platelet reactivity and platelet activation intensity, and explore the potential association with underlying inflammatory status. This was a cross-sectional cohort study carried out among PLWHIV on diverse ART regimens. Platelet reactivity and activation intensity were assessed using the bedside point-of-care VerifyNow assay, in P2Y12 reaction units (PRU), measurements of monocyte-platelet complexes, and P-selectin and GPIIb/IIIa expression increase, following activation with ADP, respectively. Levels of major inflammatory markers and whole blood parameters were also evaluated. In total, 71 PLWHIV, 59 on ART and 22 healthy controls, were included in this study. PRU values were significantly elevated in PLWHIV compared to controls [Mean; 257.85 vs. 196.67, *p* < 0.0001], but no significant differences were noted between ART-naïve or ART-experienced PLWHIV, or between TAF/TDF and ABC based regimens, similar to systemic inflammatory response. However, within-group analysis showed that PRUs were significantly higher in ABC/PI vs ABC/INSTI or TAF/TDF + PI patients, in line with levels of IL-2. PRU values did not correlate strongly with CD4 counts, viral load, or cytokine values. P-selectin and GPIIb/IIIa expression increased following ADP activation and were significantly more prominent in PLWHIV (*p* < 0.005). Platelet reactivity and platelet activation intensity were shown to be increased in PLWHIV, but they did not appear to be related to ART initiation, similar to the underlying systemic inflammatory response.

## 1. Introduction

Highly active antiretroviral therapy (HAART) has significantly increased the life expectancy of people living with human immunodeficiency virus (HIV) (PLWHIV) [1]. However, the aging of the PLWHIV population, in conjunction with the introduction of new, less toxic formulations, has unavoidably driven the frame of the discussion beyond viral suppression. Following the 90-90-90 target (90% of all PLWHIV will know their HIV status, 90% of all people with diagnosed HIV infection will receive sustained treatment, and 90% of all people receiving treatment will have viral load suppression), the 4th “90”, that of good health-related quality of life has now been set into focus [2]. Nowadays, much of the attention is drawn to the number of comorbidities that PLWHIV tend to develop over the years of their survival, and how long-term exposure to antiretrovirals may have contributed to this. 

In the era of HAART, cardiovascular (CV) disease is one of the leading causes of death in PLWHIV [3]. Early in the 2000s, observational studies first identified that PLWHIV experience relatively high rates of CV events, especially myocardial infarction (MI) incidents and other coronary heart disease manifestations [4]. Due to the novelty of combination ART (cART) at the time, and the overall uncertainty surrounding its long-term safety profile, suspicion over an association between its use and CV events was raised [5]. Later, results from the Data Collection on Adverse Events of Anti-HIV Drugs (DAD) study, the largest study to date on the effect of ART on CV events, established the increased risk of MI in people receiving cART [6]. Ever since, conflicting reports and pooled data analyses from the past decade have caused scrutiny over the issue [7,8]. Prospective cohorts have revisited the issue from different perspectives, and much diverseness may be noted in study design and result interpretation.

At the moment, common schemes of initial cART consist of a backbone regimen of a nucleotide reverse transcriptase inhibitor (NRTI) or non-NRTI in combination with a protease inhibitor (PI) or integrase strand transfer inhibitor (INSTI) [9]. Major NRTIs currently used include tenofovir disoproxil fumarate (TDF), tenofovir alafenamide (TAF), abacavir (ABC), lamivudine (3TC) and emtricitabine (FTC), while common representatives of nNRTIs are efavirenz (EFV) and doravirine (DOR). ABC has been associated with the occurrence of cardiovascular complications in many, but not all, observational studies [10,11,12,13]. Similarly, the DAD study described an association between prominent CV risk factors and the use of protease inhibitors (PIs) [14,15]. 

Moreover, emerging data on the interplay between long-standing HIV infection, chronic inflammation and thrombosis suggest a broader context of the underlying pathophysiology. Numerous sources point to the significance of increased endothelial dysfunction and platelet differential reactivity or activation intensity, as potential prothrombotic mechanisms in PLWHIV [16,17,18,19,20]. Interestingly, there are reports to suggest that different ART regimens—most commonly ABC and PIs—influence and potentially alter the expression of platelet surface receptors, thereby modifying their activation and aggregation processes [21,22,23,24,25,26]. Recent meta-analyses seem to verify the association of ABC and tenofovir-containing treatment regimens with an elevated risk of acute MIs, regardless of underlying risk factors [27,28,29]. Data on the matter remain largely heterogenous to date; however, patients with high cardiovascular risk are currently channeled away from ABC or PI use according to current guidelines [9]. This is of particular interest in the era of COVID-19, where infection with SARS-CoV-2 also increased thrombotic events, especially in high-risk populations [30]. Co-infection with SARS-CoV-2 in PLWHIV on ART is currently under investigation to assess the effects of different parameters on endothelia and further incidence of adverse cardiovascular events [31].

All that said, we hypothesized that HIV and/or ART has an impact on platelet function and the systemic inflammatory response. Hence, we aimed to explore platelet reactivity and platelet activation intensity in naïve- and treatment-experienced PLWHIV, as well as, investigate a potential association with underlying inflammatory status.

## 2. Materials and Methods

This was a cross-sectional cohort study performed at the HIV unit of a tertiary university hospital between March 2016 and December 2018. The study was conducted according to declaration of Helsinki and Good Clinical research Practice. The protocol was approved by the local research ethics committee and institutional review board (2944/12.2.2016) 

### 2.1. Patient Enrollment

Following signed informed consent, adult PLWHIV (on triple cART including NRTIs as the backbone according to current guidelines i.e., TAF, TDF, or ABC) and treatment-naïve PLWHIV (treatment naïve) attending the HIV unit of a tertiary university hospital were included in this study. Of note, patients had initiated ART upon diagnosis, as per current guidelines. Patients were excluded in the presence of pregnancy, malignancy or autoimmune disease to avoid bias of additional causes of underlying chronic inflammation. Patients receiving immunosuppressive therapy, with a recent history of bleeding within the previous three months, with an inherited (von Willebrand factor deficiency, Glanzmann thrombasthenia, Bernard-Sulier syndrome) or acquired defect of platelets (heparin induced thrombocytopenia) during the last three months prior to recruitment, with use of P2Y12 or GPIIb/IIIa receptor inhibitors, i.e., clopidogrel, prasugrel, ticangrelor, cangrelor, tirofiban, eptifibatide over the last 15 days prior to recruitment, need of hemodialysis, hematocrit > 52%, and platelets > 500 K/µL were also excluded from this study, similar to methods previously described elsewhere [32]. Healthy volunteers served as the control group. Following initial classification into groups based on backbone regimen, i.e., treatment-naïve, TAF/TDF or ABC, patients were sub-divided further depending on the combination with a third agent, i.e., PI (TAF/TDF + PI or ABC + PI) or INSTI (TAF/TDF + INSTI or ABC + INSTI).

### 2.2. Platelet Function Tests

#### 2.2.1. Platelet Reactivity

Peripheral venous blood samples were drawn in a fasting state. The first 2–4 mL of blood was discarded to avoid spontaneous platelet activation, and blood was collected in 3.2% citrate (1.8 mL draw plastic Vacuette tubes; Greiner, Monroe, NC, USA). Platelet function testing was performed with the VerifyNow (Accriva Diagnostics, San Diego, CA, USA) point-of-care P2Y12 assay based on turbidimetric optical detection of platelet aggregation in whole blood. Following sampling, citrated whole blood was transferred into a standard cartridge made of two channels. One channel contained a combination of 20 mM ADP and 22 nM PGE1. PGE1 was added to specifically suppress ADP-mediated P2Y1 platelet aggregation. The second channel contained 3.4 mM TRAP to induce platelet aggregation without going through any ADP receptor activation, as an internal reference control. As aggregation occurs, the system converts luminosity transmittance results into arbitrary units (also called P2Y12 reaction units, PRU) [32,33,34,35].

#### 2.2.2. Platelet Activation Intensity

**Figure 1:**
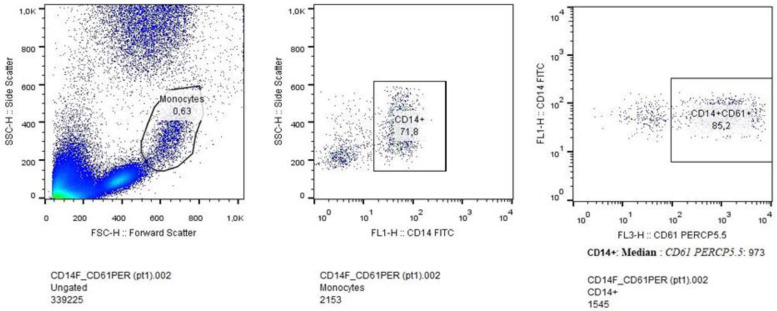
Monocyte selection process. The monocyte population was selected using flow-cytometry based on CD14 membrane receptor expression. The subpopulation forming complexes with platelets (CD61+) was then identified.

All procedures were carried out according to a previously well described protocol [36]. In brief, blood was collected in a glass sterile tube with 3.2% sodium citrate anticoagulant. Platelet and monocyte populations from whole blood samples of PLWHIV were isolated according to standard methods [37]. Immediate sample processing was performed to avoid premature platelet activation. Samples were tested by flow cytometry for the presence of monocyte-platelet complexes (MPCs) that are markers of platelet activation, as well as, for the expression rates of surface markers of platelet activation after ADP administration so as to assess the propensity of platelets to form aggregates. The platelet population was identified through the presence of the membrane receptor CD61, and then, the expression of P-selectin and the glycoprotein GPIIb/IIIa on their cell surface was demonstrated through histogram generation to determine their activation levels. Alterations in P-selectin and GP IIb/IIIa expression, acting as surrogates for platelet activation intensity and reactivity, were determined by antibody binding capacity (CD62-P and PAC-1 Abs, respectively), at baseline and following activation with ADP. Monocytes were identified based on the membrane receptor CD14 and separated from other cells based on size and granulation (forward vs. side scatter). CD61+ cells were identified within the CD14+ population to investigate the propensity to form MPCs. MPC isolation as per the protocol is displayed in Figure 1.

All antibodies were supplied from Becton Dickinson. ADP was produced by Sigma-Aldrich under company code 01905-250MG-F and lot no. BCBP6676V. Red cells were lysed by adding 500 µL of lysis buffer with cat no. 555899 and incubated for 10 min at room temperature in the absence of light. After checking the clarity of the cells as an indication of their successful lysis, washing was performed by centrifugation for 5 min at 4 °C at 2000 rpm. The supernatant was removed and 300 µL of PBS was added to maintain cell viability until subsequent treatment. 

### 2.3. Assays for Cytokine Measurement

Blood was collected by venipuncture from a peripheral vein into BD Vacutainer SSTII Serum Separator Tubes (cat#367955). The tubes were gently inverted 5–6 times, left to clot for 30 min at room temperature and centrifuged for 10 min at 1600× *g* at RT. The serum was aliquoted and stored at −70 °C until needed.

Measurement of the concentration of the cytokines IL-2, IL-4, IL-6, IL-10, IL-17a, IFNγ and TNF in patient serum samples was performed with a BD FACS Array Bioanalyzer, using a cytometric bead array (CBA) assay (Human Th1/Th2/Th17 Kit, cat#560484, BD Biosciences, San Diego, CA, USA) (range 20–5000 pg/mL for all analytes, sensitivity 2.6 pg/mL for IL2, 4.9 pg/mL for IL4, 2.4 pg/mL for IL6, 4.5 pg/mL for IL10, 18.9 pg/mL for IL17a, 3.7 for IFNγ and 3.8 pg/mL for TNF).

### 2.4. Statistical Analysis

Categorical data are presented as group percentages and frequencies. Continuous data with normal and skewed distribution are presented as mean ± SE and medians (first to third quartiles), respectively. The Fisher exact test and two-sample t-test were used for comparison of normally distributed categorical and continuous data, respectively. The Kruskal Wallis test was used for comparison of non-normally distributed data. Data normality was assessed using the Shapiro-Wilk tests using an a of 0.05. Bonferroni post hoc correction was used to assess multiple comparisons across groups. Correlations were examined using Spearman’s rank correlation coefficients. All tests were two-tailed and statistical significance was considered for *p*-values < 0.05. Analyses were performed using SPSS for Windows (version 24.0 SPSS IBM Inc., Chicago, IL, USA).

## 3. Results

In total, 93 subjects were included in this study (71 PLWHIV and 22 healthy controls), following implementation of inclusion/exclusion criteria. Seventy-one had been receiving cART consistently for at least three months, of whom 38 received TAF or TDF, and 21 received ABC, as a backbone NRTI-based regimen. Twelve treatment-naive PLWHIV and a group of 22 HIV negative individuals served as healthy controls. Population characteristics of each subgroup are displayed coherently in Table 1. Groups were comparable except for viral load (*p* = 0.039), as expected. Differences were driven by significant differences between ART naïve and treated patients (*p* = 0.015 for TAF/TDF and *p* < 0.001 for ABC).

**Figure 2:**
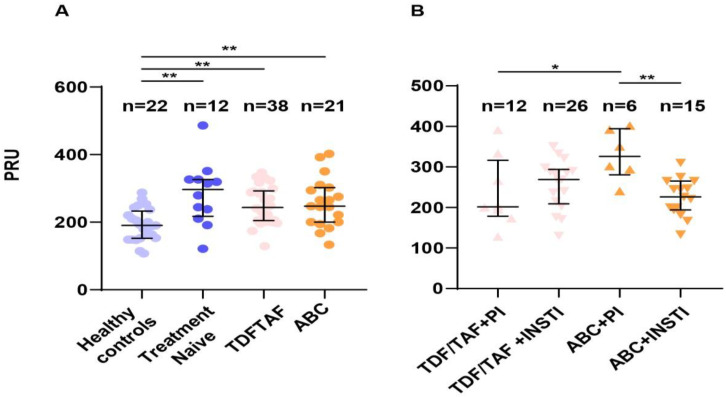
Platelet reactivity measurements (**A**). Platelet reactivity was significantly increased in PLWHIV compared to healthy controls. No differences were noted within treatment groups (**B**). PLWHIV receiving the combination of ABC + PI presented significantly increased platelet activation compared to patients on TDF/TAF + PI or ABC + INSTI. TAF: tenofovir alafenamide, TDF: tenofovir disoproxil fumarate, ABC: abacavir, PI: protease inhibitor, INSTI: integrase strand transfer inhibitor; PRU: platelet reactivity units; ** denotes *p* < 0.01, * *p* < 0.05.

Inferential analysis revealed that PRU values were significantly elevated in PLWHIV compared to controls [mean; 257.85 vs. 196.67, *p* < 0.001], either treatment-naïve or on therapy, but no significant difference was noted between TDF/TAF and ABC groups (Table 1 and Figure 2A). Subgroup analysis revealed an increased PRU in patients receiving the combination of ABC + PI comparing to those receiving TDF/TAF + PI or ABC in combination with an integrase strand transfer inhibitor (INSTI)(ABC + INSTI) (*p* < 0.05 and *p* < 0.01 respectively)(Figure 2B). Females proved to have significantly higher PRU measurements than males [mean; 298.18 vs. 243.79, *p* = 0.001]. PRU values did not significantly correlate with CD4 counts or detectable viral load.

**Figure 3:**
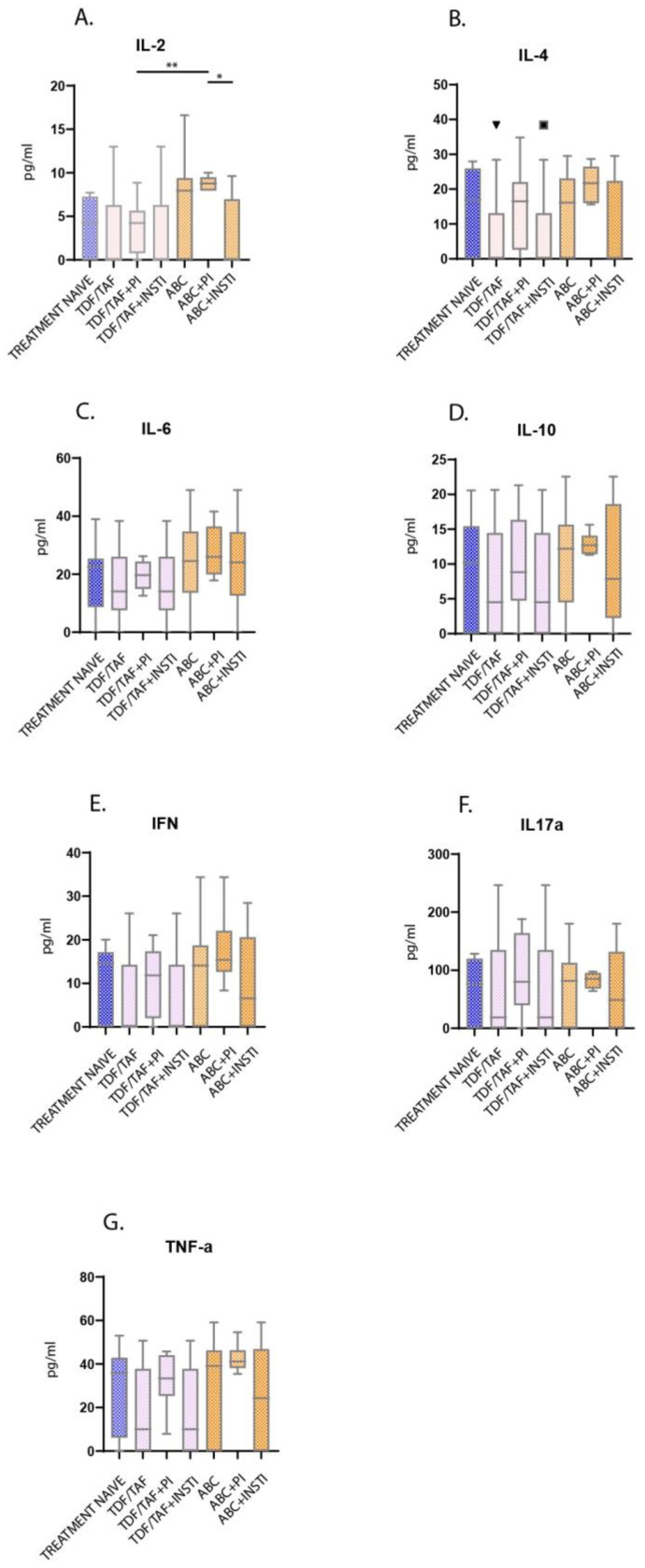
Inflammatory response assessment, as reflected in major pro- and anti-inflammatory mediator levels (**A**–**G**). Tukey box plot reveals significantly elevated levels of IL-2 in patients receiving combination of ABC + PI compared to patients on TDF/TAF + PI or ABC + INSTI. IL: Interleukin, TNF: tumor necrosis factor, IFN: interferon, TAF: tenofovir alafenamide, TDF: tenofovir disoproxil fumarate, ABC: abacavir, PI: protease inhibitor, INSTI: integrase strand transfer inhibitor; PRU: platelet reactivity units; ** denotes *p* < 0.01, * *p* < 0.05.

No significant differences in cytokine levels were detected among treatment groups (Table 1) (Figure 3A–G), except for IL-2 levels where a significant proportion of pro-inflammatory cytokine was detected in patients receiving ABC + PI comparing to those receiving TDF/TAF + PI or ABC + INSTI (*p* < 0.01 and *p* < 0.05, respectively) (Figure 3A). No significant differences were recorded in IL-6 (*p* = 0.08), IL-10 (0.09) and TNF (0.61) levels between healthy subjects and treatment-naïve PLWHIV (Table 1).

In the control group, PRU levels adversely correlated with IL-6 levels (rho = −0.715, 95% CI: −0.897, −0.325, *p* = 0.002). In treatment-naive PLWHIV, PRUs negatively correlated to IFN (rho = −0.656, 95% CI: −0.914, −0.24, *p* = 0.039), IL-2 (rho = −0.632, 95% CI: −0.906, 0.018, *p* = 0.05), IL-6 (rho = −0.77, 95% CI: −0.945, −0.251, *p* = 0.009), IL-10 (rho = −0.755, 95% CI: −0.941, −0.217, *p* = 0.01), IL-17A (rho = −0.742, 95% CI: −0.938, –0.191, *p* = 0.014) and TNF-a (*p* = −0.699, 95% CI: −0.926, −0.102, *p* = 0.024) levels. In people treated with TDF/TAF, PRU correlated with white blood cell counts (rho = −0.365, 95% CI: −0.640, −0.008, *p* =0.04) in people treated with ABC with LDL-C levels (rho = −0.537, 95% CI: −0.814, −0.06, *p* =0.02). Potential correlations with hemoglobin values were not taken into consideration since they represent a commonly reported laboratory interference [38].

PLWHIV receiving either ABC or TDF/TAF do not display a statistically significant difference in the formation of monocyte-platelet complexes (MPCs), suggesting that platelets form complexes with monocytes at the same rate, regardless of the drug being tested (*p* = 0.215). At the same time, the formation of MPCs in PLWHIV, as compared to that of a healthy population, utilizing historic data from Loguinova et al. was significantly higher (*p* < 0.05) [39]. 

**Figure 4:**
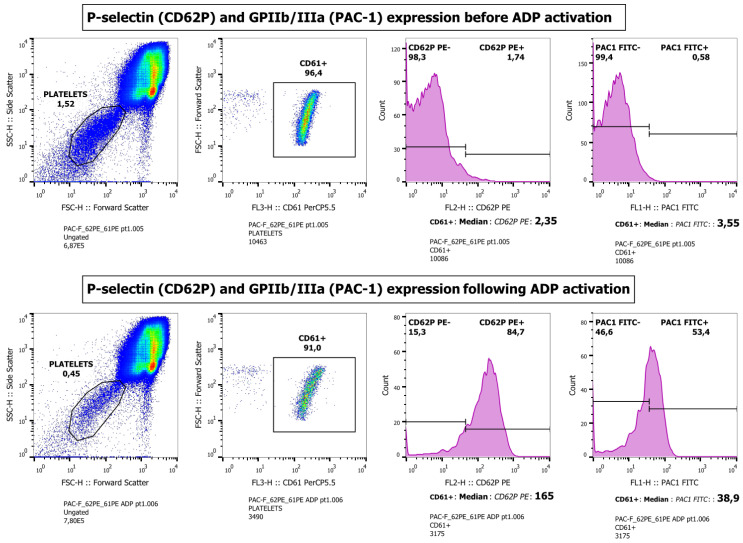
Platelet activation intensity assays. Platelets were studied both at rest and following ADP activation. Platelet population was initially identified via CD61 membrane receptor, and then histogram formation revealed membrane P-selectin and glycoprotein GPIIb/IIIa to assess their activation. Non-activated platelets at rest (**upper panel**) expressing membrane receptor CD61 were initially isolated. Consequently, subpopulations expressing surface CD62P and PAC-1 were identified, respectively. A similar process was carried out following ADP activation (**lower panel**).

P-selectin and GP IIb/IIIa expression (denoted as median fluorescent intensity), reflecting platelet activation intensity, significantly increased following ADP activation, as revealed by the prominent difference in their surrogate marker antibodies’ selection (*p* < 0.05 for CD62-P and PAC-1, respectively) (Table S1). The isolation of activated platelet populations before and after ADP induction may be seen comparatively in Figure 4 and Table S1. However, no difference was detected in ADP-induced activation levels between the antiretroviral treatment subgroups tested (Table S1).

## 4. Discussion

In the present study, we have shown increased P2Y12-mediated platelet reactivity and platelet activation intensity in PLWHIV, compared to healthy subjects. Both phenomena were comparable between ART-naïve or -experienced PLWHIV, while in the latter case they did not seem to significantly differ between TDF/TAF and ABC based treatment regimens. However, a significantly higher PRU was recorded in ABC/PI compared to ABC/INSTI or TAF/TDF + PI groups. Our findings did not demonstrate an association with underlying systemic inflammatory responses, as reflected in the levels of pro- and anti-inflammatory mediators; however, significantly increased levels of IL-2 have been detected in patients receiving the combination of ABC and PI, compared to TDF/TAF and PI or ABC and INSTI.

The immune system of PLWHIV is constantly activated, resulting in the accumulation of cells other than platelets at the site of the lesion, thus exacerbating the inflammatory situation. Chronic inflammation, immunodeficiency/reduced CD4 counts, endothelial dysfunction, increased thrombosis and accelerated atherosclerosis are among the phenomena that have been associated with the occurrence of cardiovascular events in both PLWHIV and healthy individuals [40,41,42,43]. The high prevalence of lifestyle factors, such as dyslipidemia and smoking [44], however, makes PLWHIV more susceptible to CV disease. At the same time, the use of certain antiretrovirals, as well as, the ongoing state of inflammation may contribute to increased endothelial dysfunction and hypercoagulability [45]. The mechanisms linking immune activation, inflammation and the increased rate of thrombotic cardiovascular complications have yet to be fully explored in HIV. Nonetheless, platelet-mediated thrombogenesis shows much promise in all respects.

We aimed to test the hypothesis of HIV- and ART-related immune thrombosis via assessment of platelet function and underlying inflammatory response. Our findings are in agreement with a recent meta-analysis assessing 30 studies and including 2325 participants [46], evaluating platelet activation in PLWHIV. However, even though, in the latter study platelet activation was assessed with various techniques, platelet function using light transmission aggregometry, as per our report, was only assessed in two studies, while significant heterogenicity between molecules measured and techniques employed for assessment of platelet activation was noted [46]. We showed significantly increased platelet activation in PLWHIV, compared to healthy individuals. A possible explanation lies in the fact that HIV infection per se might drive increased platelet reactivity or activation intensity through chronic inflammation or other signaling pathways [46]. Despite this, available regimens including PIs are highly potent, and hence the case of inadequate viral suppression and ongoing hyper-inflammation is unlikely. As also reflected in the non-detectable viral load of this group of patients (data not shown), a residual inflammatory response is possible. Persistence of HIV viral reservoirs, intestinal microbial translocation and co-infections may contribute to this state of events [47].

ART has increased the life expectancy of PLWHIV, exposing them to conditions that older people face, such as cardiovascular disease (CVD). The Veterans Ageing Cohort Study (VACS) reported a higher rate of acute MI in PLWHIV than in healthy individuals, having excluded people with baseline CVD and accounted for traditional risks such as smoking and dyslipidemia [42]. In this study, among veterans living with HIV, neither baseline viral load nor CD4 cell count nor ART class or regimens were associated with increased odds of acute MI [42]. However, at the time of the analysis, less than half of PLWHIV had started ART, and a borderline association with recent PI exposure was detected, thus hinting at the idea that ART enhances the risk of MI [42]. Similar reports by Durand et al. and Desai et al. produced findings in agreement with other authors, raising the interest to further investigate the effect of specific ART drugs on the pathogenesis of cardiovascular complications in PLWHIV [48,49]. 

We showed that platelet reactivity in PLWHIV remains unchanged following ART initiation, in line with a number of previous authors that have also reported persistently activated elevated levels of platelet reactivity within 6–24 months of initiating treatment [41,50,51,52,53]. Previous observations have reported that no consistent differences in platelet reactivity were seen in ART-naïve PLWHIV compared with ART-treated patients, suggesting that ART as a whole does not increase but also does not attenuate platelet reactivity [54]. However, it is intriguing that in patients receiving PIs—and despite successful viral suppression—activation does not seem to be attenuated, as in the case of non-PI containing regimen recipients [52,55,56,57,58]. In fact, the levels of platelet activation seem to be 2-fold higher in patients on PI-based therapy compared to treatment-naïve PLWHIV [50,51,59].

Moreover, our study demonstrated that platelet activation does not seem to significantly differ between treatment groups including TDF/TAF or ABC, despite data from the DAD study highlighting the use of ABC in the antiretroviral regimen as being of maximal importance for the development of acute MI incidents [60]. Our data are consistent with the results of a recent report, in which in vitro incubation of platelets with ABC and TDF displayed no difference in platelet aggregation upon activation [61]. Similarly, a recent crossover trial involving PLWHIV treated with ABC and TDF revealed no significant alterations in primary or secondary hemostatic pathways, as assessed by thromboelastography. However, switching to ABC treatment shifted multiple biomarkers, including platelet reactivity and proinflammatory markers, to a pro-coagulant direction, thereby suggesting a multifaceted mechanism in the development of thrombotic risk [62].

Even though our sample size was small following subgroup analysis, a significant difference in PRU levels was detected across specific treatment subgroups i.e., ABC/PI vs TAF/TDF + PI or ABC/INSTI. In the former case (ABC/PI vs. TAF/TDF + PI), it is likely that the combination of these regimens (ABC/PI) poses an additive to platelet activation, while in the latter (ABC/PI vs. ABC/INSTI), PI parameters could drive final measurements. Aberrant purinergic signaling has been implicated in ABC-induced endothelial dysfunction [63], while ABC enhanced collagen-evoked platelet aggregation in vivo via platelet aggregation, and interrupted NO-mediated platelet inhibition [21,61,64]. ABC and PIs are described to have an independent effect on the risk for CV events “above their potential metabolic effects” [10]. This is in line with respective findings showing that IL-2 is increased in these groups of patients. In the case of ABC + PI where significant differences were detected, the case might be that underlying inflammatory response—via pharmacologic enhancement—is one of the main causes. It is possible that the combination of both regimens, and not each on its own, exerts proinflammatory activation. Besides, no relation of ABC with inflammatory response has been shown in the past [23,65]. However, due to small number of patients results, this should be interpreted with caution. No relation of ABC with inflammatory response, i.e., IL-6 levels, has been shown in the past [23,65], while TAF/TDF equally seem to attenuate inflammatory response [66].

Utilizing flow cytometry methods, we analyzed the biological activity of platelets immediately after blood collection and tested the tendency of platelets to form complexes with monocytes, as well as the reactivity of platelets after induction of activation, in PLWHIV. Platelet monocyte aggregates were found to be increased in our population of PLWHIV, but did not significantly differ between treatment groups. Previous studies have confirmed an increase of PMCs in treatment-naïve individuals [67]; however, a decrease was observed in patients receiving raltegravir compared to patients receiving NNRTIs or PI-containing regimens [52]. The activated platelets bind via P-selectin to the PSGL-1 receptor on monocytes, forming aggregates and approaching damaged endothelial sites. At the same time, activated platelets release thrombotic factors and substances that further promote the activation of adjacent platelets and their aggregation. 

In addition, P-selectin expression is tied to the process of platelet degranulation that has been shown to be defective in PLWHIV, leading to persistent platelet activation [68]. Data concerning soluble and non-soluble P selectin in PLWHIV pre- and post- ART initiation have been contradictory. Even though most—but not all—studies have found increased levels in treatment-naïve individuals, it seems that platelet enhancement is equally preserved, even following ART [41,50,57,68,69]. However, other authors have not shown a significant impact of cART on monocyte, endothelial and platelet function [54,55,56,70]. Nonetheless, the case of persistence of increased soluble and non-soluble P selectin levels did not necessarily come with increased risk of adverse cardiovascular events [57]. It is likely that differences in the treatment regimens between different studies account for the incongruent effect estimates [46]. Overall, our findings highlight the potential for enhanced platelet activation and monocyte complex formation in PLWHIV and are in accordance with previous observations on the functionality of platelets in this population [17,41,69], although no association with underlying inflammatory response was shown. 

A number of limitations are identified in this study. First of all, platelet reactivity was largely assessed via ADP stimulation of the P2Y12 receptor, P selectin expression and MPCs. It remains unknown whether other pathways or molecules of platelet stimulation assessment e.g., CD40 ligand [51,59,71], RANTES chemokine ligand [59,72,73,74] etc., result in similar reactivity or differentiate among various ART regimens. Moreover, our exclusion criteria were quite strict, excluding a broad spectrum of immunocompromised patients, in order to avoid bias during evaluation of underlying inflammatory response. Even though in the real-world cART remains highly potent so that PLWHIV live non-compromised lives, it is possible that different co-morbidities do affect the impact of therapy on chronic inflammation, and thus platelet activation and reactivity. When a cure is finally achieved, many of our questions are bound to be answered, since the design of a cross-sectional study looking into effects before HIV infection, while on disease and following cure beyond viral suppression could reliably investigate the potential restoration of platelet activation and differentiate possible causes in this group of patients, similar to other diseases [75,76]. Nonetheless, in the experience of these authors, frequent visits and recurrent blood sampling discourage patient enrollment, especially in applied basic research studies as our own. The population remains limited due to strict exclusion criteria in combination with variable patient compliance to therapy and attendance visits. Even though sample size allowed for statistical significance to be detected, it would be interesting to expand the study population to more than one center, permitting multivariable analysis and examining associations of 3rd regimens within different cART groups, as well as currently recommended dual therapy. Last, in the context of diverse pharmacological properties between TAF and TDF, and their respective impact on metabolic profiles that pose an effect on cardiovascular risk, it would be useful to further look into this subgroup of patients, even though current data did not confirm any difference pertaining to platelet reactivity [61]. In the same context, within-class differences, e.g., PIs, would be worth studying since their impact on metabolic profiles remains diverse [77].

## 5. Conclusions

HIV infection per se, as well as ART, have been associated with increased thrombotic risk, leading to adverse cardiovascular events. Underlying mechanisms driving this phenomenon remain elusive, while the role of platelets is currently under investigation. We found increased platelet activation and intensity in PLWHIV, even though platelet activity as tested here does not seem to significantly differ between TAF/TDF and ABC based combinations, pushing focus to other pathways and mediators. In this setting, one would be worth wondering whether in PLWHIV with no increased cardiovascular risk, but still elevated platelet activation, the use of antiplatelets would be of benefit, as in acute bacterial infection [78]. Or, if an increased dose or use of more potent antiplatelet regimens would be necessary in patients with history of cardiovascular disease or receiving PIs, in view of recent findings of high on treatment platelet reactivity in PLWHIV [79]. Whether platelet reactivity and activation intensity could in the future represent a reliable surrogate marker for major adverse cardiovascular events remains to be seen.

## Figures and Tables

**Table 1:** Population characteristics.

	Healthy Controls (*n* = 22)	ART Naive (*n* = 12)	Abacavir (*n* = 21)	Tenofovir (*n* = 38)	*p*
Male *n* (%)	14 (63.6)	10 (83.3)	15 (71.4)	28 (73.7)	0.130
Age, median (minimum-maximum)	48.5 (25–72)	36.5 (20–52)	42 (29–65)	43 (21–76)	0.180
Co-infection *n* (%)					
HBV (+)	----	2 (16.7)	1 (4.8)	6 (15.8)	0.540
HCV (+)	----	2 (16.7)	0 (0.0)	8 (21.1)	0.055
CCI, median	1 (0–1)	0 (0–1)	0 (0–1)	1 (0–2)	0.090
3rd agent *n* (%)	----				0.810
INSTI	----	----	15 (71)	26 (69)	
PI	----	----	6 (29)	12 (31)	
CD4 (cells/mm^3^), median (IQR)	----	324 (54–798)	702 (503–1065)	575 (414–726)	0.057
Viral load (copies/mL), median (IQR)	----	64,000 (0–710,000)	0 (0–108,000)	0 (0–53,800)	0.039
WBC (/mm^3^), median (IQR)	6700 (5500–8000)	6070 (5082–8037)	7370 (6225–8812)	6550 (5345–7975)	0.193
Hb (g/dL), median (IQR)	14 (13.25–14)	13.6 (9.9–15.45)	14.35 (13–15.6)	15 (13.4–15.7)	0.050
PLTs (k/mm^3^), median (IQR)	253 (206–279)	190 (156–239)	236 (195–255)	230 (199–267)	0.400
eGFR (mL/min/1.73 m^2^)	98 (74–130)	100 (93–117)	95.5 (78–109)	96 (80–110)	0.280
Total Cholesterol (mg/dL), median (IQR)	181 (159–240)	188 (162–222)	200 (176–231)	182 (154–202)	0.100
LDL-C (mg/dL), median (IQR)	108 (103–133)	116 (112–126)	111 (85–145)	103 (89–130)	0.790
HDL-C (mg/dL), median (IQR)	45 (40–60)	46 (40–56)	52 (42–61)	49 (42–55)	0.230
TRG (mg/dL), median (IQR)	120 (77–196)	146 (71–236)	131 (72–217)	114 (86–172)	0.230
PRU, median (IQR)	190 (160.5–240)	296.5 (217–326)	245 (200–298)	243.5 (200–296)	0.005
IFN-α (pg/mL), median (IQR)	----	14.61 (0–17.1525)	14.08 (0–18.700)	8.908 (0–17.7)	0.510
IL-2 (pg/mL), median (IQR)	----	4.264 (0- 7.277)	7.952 (0–9.3940)	3.218 (0–7.494)	0.440
IL-6 (pg/mL), median (IQR)	6.86 (1.56–16.2)	23.265 (10.87–28.9)	24.52 (13.66–34.73)	19.04 (12.67–25.88)	0.740
IL10 (pg/mL), median (IQR)	3.18 (2.3–13.23)	10.167 (0–15.425)	12.19 (4.5–15.63)	8.9125 (4.5–16.565)	0.820
TNF (pg/mL), median (IQR)	26.37 (4–155.9)	39.950 (6.21–42.86)	39.09 (0–46.230)	27.475 (0–44.045)	0.520

HBV; hepatitis B virus, HCV; hepatitis C virus, INSTI; integrase strand transfer inhibitors, PI; protease inhibitors, ART; antiretroiral therapy, NRTIs; nucleoside reverse transcriptase inhibitors, IQR; interquartile range, PRU; P2Y12 reaction units, WBC; white blood cells, Hb; hemoglobin, PLTs; platelets, eGFR; estimated glomerular filtration rate, LDL-C; low-density lipoprotein, HDL-C; high-density lipoprotein, TRG; triglycerides, IFN; interferon, IL; interleukin, TNF; tumor necrosis factor, CCI: Charlson’s comorbidity index.

## Data Availability

The data presented in this study are available on request from the corresponding author.

## Supplementary Materials

The following supporting information can be downloaded at: https://www.mdpi.com/article/10.3390/microorganisms11040958/s1, Table S1. Platelet activation assays.
