# Supplementary material for: Association of Antiretroviral Therapy with Platelet Function and Systemic Inflammatory Response in People Living with HIV: A Cross-Sectional Study
Source: Microorganisms. 2023 Apr 6;11(4):958. doi: 10.3390/microorganisms11040958 (PMC10144397; doi:10.3390/microorganisms11040958)
Supplement: Supplementary file 1 [file microorganisms-11-00958-s001.zip › microorganisms-2158799-supplementary.pdf]

Table S1. Platelet activation assays.

	TDF	TAF	ABC	Statistical Significance (*p*)
**Prior to ADP activation**				
CD62P	3.318	3.008	3.792	0.765
PAC-1	3.624	3.680	6.858	0.174
**Following ADP activation**				
CD62P	103.30	105.00	165.833	0.326
PAC-1	30.460	28.586	33.650	0.930
Values express median fluorescent intensity (MFI)				
